# Multimethod to prioritize projects evaluated in different formats

**DOI:** 10.1016/j.mex.2021.101371

**Published:** 2021-05-01

**Authors:** Felipe Diniz Ramalho, Iara Sibele Silva, Petr Y. Ekel, Carlos Augusto Paiva da Silva Martins, Patrícia Bernardes, Matheus Pereira Libório

**Affiliations:** a Pontifical Catholic University of Minas Gerais, Belo Horizonte, MG 30535-610, Brazil; b Asotech-Advanced System Optimization Technologies Ltda, Belo Horizonte, MG 30535-630, Brazil; c Açolab-ArcelorMittal, Nova Lima, MG 34006-042, Brazil; d Federal University of Minas Gerais, Belo Horizonte, MG 31270-901, Brazil

**Keywords:** Preference formats, Transformation functions, Portfolio of Rd&I projects, Analytic hierarchy process

## Abstract

The prioritization of Research, Development & Innovation Projects is an essential step in the innovation management process. As a rule, it is carried out applying methods that allow one to process experts' preferences concerning each project according to established criteria. However, there are different preference formats which experts can utilize: Ordering of Alternatives, Utility Values, Multiplicative Preference Relations, Fuzzy Estimates, Fuzzy Preference Relations, etc. Wherein, each prioritization method usually handles only one of these formats. Thus, the following question arises: how do we prioritize projects taken from portfolios evaluated in different formats? The proposed methodology presents a way to overcome this gap by achieving three main objectives. First, develop techniques that make it possible to crossover between preference formats and prioritization methods. Second, merge two portfolios of projects built applying different prioritization methods. Third, prioritize projects evaluated using different formats. The results of this study are universal and can be applied to replace any method of prioritization. In the specific case, the Mapping method is replaced by the Analytic Hierarchy Process and, then, by the Interactive Multicriteria Decision Making method (so called TODIM method). Techniques are also proposed to ensure compatibility between different preference formats and prioritization methods.

• Prioritization of projects evaluated in different formats using the Mapping, AHP, and TODIM methods.
• Providing fully consistent evaluation matrices.
• Application of techniques to make different preference formats and prioritization methods compatible.

## Graphical abstract

**Figure:**
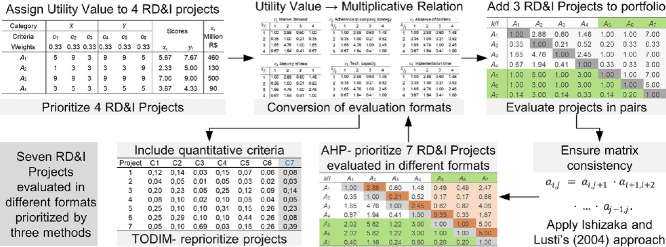

Specifications table

**Table:** 

Subject Area:	Engineering
More specific subject area:	*Project management, Decision making*
Method name:	*Multimethod to prioritize projects evaluated in different formats*
Name and reference of original method:	*Analytic Hierarchy Process and TODIM method (an acronym in Portuguese of Interactive and Multicriteria Decision Making)*
Resource availability:	*If applicable, include links to resources necessary to reproduce the method (*e.g.*, data, software, hardware, reagent)*

## Method details

### Background

The AHP is a method that permits one to decompose, organize, and analyze complex problems of multi-attribute decision-making [1]. Since its inception, AHP has become widely applied because of its simplicity and ease of deployment. It has been transformed into a popular tool for prioritization and selection of portfolios of projects [2]. The main objective of its application is to construct a ranking of alternatives by decomposing the problem under analysis into a hierarchy of criteria, sub-criteria, and alternatives [3]. Due to its reliability and accuracy characteristics, AHP has become the "most extensively used method" to prioritize projects [4], p.3.

The TODIM method (an acronym in Portuguese of Interactive Multicriteria Decision Making) is a discrete multicriteria method based on Kahneman and Tversky's prospect theory [5,6]. According to this theory, humans prefer a small gain to a larger gain associated with a specific risk of loss [5]. Gomes [7] indicates that TODIM has five main characteristics. First, it is accessible to professionals without a solid analytical background in Multiple-criteria decision-making. Second, it offers an orderly classification from which a decision can be make. Third, it allows working with both quantitative and qualitative criteria. Fourth, it ranks the criteria hierarchically. Fifth, it permits the consideration of the interdependence between the criteria. These characteristics make TODIM and its extensions particularly useful and popular for prioritizing projects [6], [7], [8], [9].

Unlike AHP and TODIM, other methods such as the Mapping method [10] proved inefficient in prioritizing projects [11]. The Mapping method consists of evaluating and plotting projects in a Bubble Chart from a set of criteria, allowing the visualization of each project's position in the corresponding portfolio [10]. The Bubble Chart is easy to implement and, therefore, it is very popular among companies [12]. In Brazil, forty-three companies have adopted the Mapping method [13]. These companies have dozens of projects evaluated by experts in their portfolios (e.g., [14]).

The problem of replacing one method of prioritization with another leads to the loss of the evaluations which have been carried out. This loss may occur because the evaluations performed by the companies may not be compatible with the AHP and TODIM method's format. In the case of AHP, evaluations of RD&I Projects in the Ordering of Alternatives (OA), Utility Values (UV), and Fuzzy Estimates (FE) formats [3] may be lost because the AHP-compatible format is Multiplicative Relations (MR) [15]. The transformation functions [3] allow one to convert preference formats and prioritize projects using different methods (e.g., Mapping, Simple Additive Weight (SAW), TOPSIS, ELECTRE, AHP, and PROMETHEE [4,10]).

Taking the above into account, it should be emphasized that the "*Multimethod to prioritize projects evaluated in different formats*" provides the replacement of inefficient prioritization methods (such as the Mapping method, for instance) with AHP and, later, by TODIM without losing the existent evaluations.

**Fig. 1:**
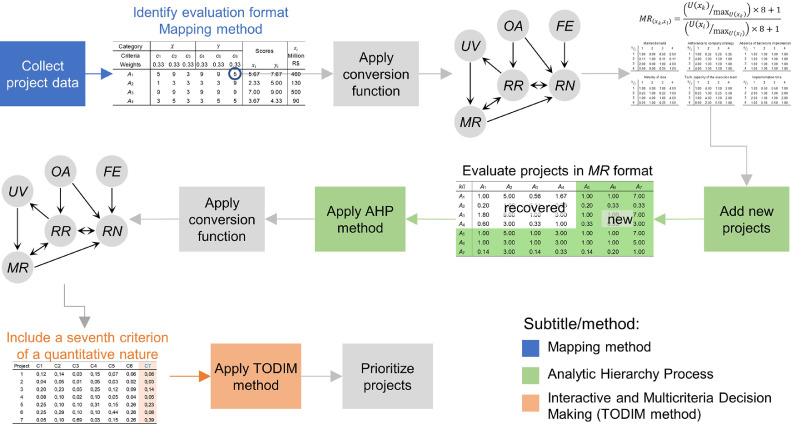
Application example of the “Multimethod to prioritize projects evaluated in different formats”.

In particular, First, the evaluations of Projects are collected and their preference format is selected [3]. Second, the transformation functions discussed in [15] are applied to convert the evaluations to the AHP format [1]. Third, new projects are added to the portfolio; these projects are evaluated in the AHP preference format and then prioritized. Fourth, the corresponding transformation functions [15] are again applied to convert the evaluations to the format of the TODIM method [6,7]. Fifth, a seventh criterion of a quantitative nature is inserted in the decision matrix. Sixth, Projects are prioritized by applying TODIM. Fig. 1 illustrates how this new method works.

## Method validation

The validation of the "*Multimethod to prioritize projects evaluated in different formats*" is performed as part of the analysis of the following problem. A company needs to replace the Mapping method with the AHP to prioritize its new three Projects without losing the evaluations of other four projects performed earlier in the UV format. Thus, it is necessary to prioritize the portfolio of seven projects. Finally, the company decides to replace the prioritization method again. This time, they want to incorporate quantitative criteria, as well as the notion of risks when prioritizing its projects applying the TODIM method.

### Mapping method

Following the work of Cooper et al. [12] and Bagno and Faria [16], the Bubble Chart can be prepared in three steps. First, the experts are to assign weights for two groups of criteria ($p_{j_{x}},j_{x}=1,\;2,\;3,\;\text{and}\;p_{j_{y}},j_{y}=1,\;2,\;3$, respectively). Second, the experts evaluate the projects using the Utility Value (UV) format through the {1,3,7,9} scale. The absence of the value 5 allows differentiating incremental from disruptive RD&I Projects [16]. In this problem, four projects were evaluated according to the criteria presented in Table 1.

**Table 1:** Evaluation criteria.

Group	Criterion	Description	Polarity
X	c1	Market demand	positive
	c2	Adherence to company strategy	positive
	c3	Absence of barriers to implementation	positive
Y	c4	Maturity of idea	positive
	c5	Technical capacity of the execution team	positive
	c6	Implementation time	negative

*Note:* The criteria of group X represent opportunity for the company. The criteria of group Y represent capacity of project execution by the company. Polarity represents the correlation between the criterion and the prioritization objective. It is positive when the criterion is positively correlated with the prioritization objective. At the same time, Polarity is negative when the criterion is negatively correlated with the prioritization objective.

Third, the score of each project is calculated for the "Opportunity" and "Capacity" groups as follows:

(1) $$x_{i}=\frac{\sum_{j_{x}=1}^{3}c_{j_{x}i}p_{j_{x}}}{\sum_{j_{x}=1}^{3}p_{j_{x}}},\;i=1,2,\ldots ,n,$$

(2) $$y_{i}=\frac{\sum_{j_{y}=1}^{3}c_{j_{y}i}p_{j_{y}}}{\sum_{j_{y}=1}^{3}p_{j_{y}}},\;i=1,2,\ldots ,n.$$

In (1) and (2), $x_{i}$ and $y_{i}$ are the weighted averages of the scores for the Group "Opportunity" and the Group "Capacity", respectively; $c_{j_{x}i}\;$and $c_{j_{y}i}$ are scores obtained by the i-th Project for the criteria $j_{x}$ and $j_{y}$, respectively. Fourth, the estimated Cost of the Project development is attributed to the third ordinate $z_{i}$.

Fifth, the estimates (1) and (2) are used as the ordered pairs $x_{i},y_{i}$ while $z_{i}$ is used to define the bubble size on the chart. Table 2 shows the expert evaluations for a portfolio with four Projects.

**Table 2:** Evaluation matrix of projects in UV.

Group	X			Y			Scores		$z_{i}$ Million R$
Criteria	*c*_1_	*c*_2_	*c*_3_	*c*_4_	*c*_5_	*c*_6_	$x_{i}$	$y_{i}$	
Weights	0.33	0.33	0.33	0.33	0.33	0.33			
*A*_1_	5	9	3	9	9	5	5.67	7.67	460
*A*_2_	1	3	3	3	3	9	2.33	5.00	130
*A*_3_	9	9	3	9	9	9	7.00	9.00	500
*A*_4_	3	5	3	3	5	5	3.67	4.33	90

*Note:* The weights for all six criteria are equal. The $x_{i}$ scores are obtained by applying (1) The $y_{i}$ scores are obtained by applying (2). The value of $z_{i}$ represents the Cost of a Project.

**Fig. 2:**
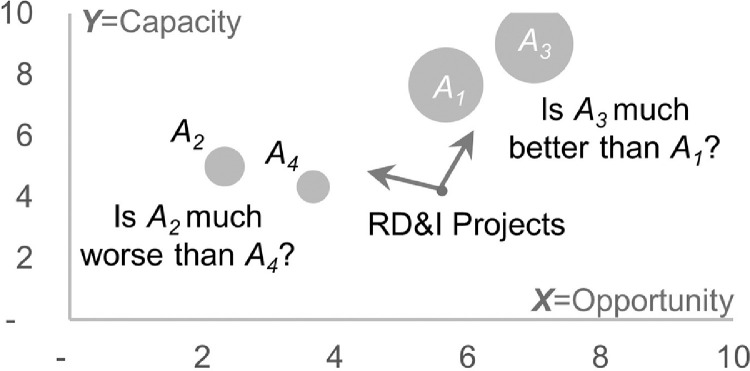
The bubble chart built from the data of Table 2. The circles in Fig. 4 represent the four Projects evaluated in the UV format. The size of the circles reflects the values of $z_{i}$.

Table 2 includes all the data necessary for the construction of the Bubble Chart. Fig. 2 illustrates the Bubble chart elaborated with the scores obtained applying (1) and (2) and the $z_{i}$ values.

Fig. 2 shows that the prioritization of Projects by the Mapping method is not very informative even for a portfolio of only four Projects. The generated result does not allow one to determine how much one project is better or worse than another. However, before replacing the Mapping method with AHP, it is necessary to convert the evaluations already carried out. In particular, it is necessary to convert the evaluations in UV format to the Multiplicative Relations (MR) format.

### Conversion of evaluations in UV format to MR format

The conversion of experts' evaluations from the UV format to the MR format was carried out by adapting the following transformation function proposed in Herrera et al. [17]:

(3) $$MR_{\left(x_{i},x_{l}\right)}=\frac{u_{i}^{k}}{u_{l}^{k}}$$

Basically, the utility function U(X)→[0,1] is normalized for the interval [1,9] before applying the function (3) of [17]. The adapted transformation function that converts evaluations to MR from $UV\;=\;\{u_{i}\},i\;=\;1,2,\ldots ,n$ is the following:

(4) $$MR_{\left(x_{k},x_{l}\right)}=\frac{\left(U\left(x_{k}\right)\;/\;\text{ma}x_{U\left(x_{k}\right)}\right)\times \;8+1}{\left(U\left(x_{l}\right)\;/\;\text{ma}x_{U\left(x_{l}\right)}\right)\times \;8+1},\;k,l\;=\;1,2,\ldots ,n$$

where 8 is the difference between the limits of the Saaty scale [1].

The conversion function (4) allows transforming UV→MR. The results of this transformation are illustrated in Table 3.

**Table 3:** Representation of criterion *c*_1_ to *c*_6_ through *MR*.

*c*_1_ Market demand				
*k*/*l*	1	2	3	4
1	1.00	2.88	0.60	1.00
2	0.35	1.00	0.21	0.35
3	1.65	4.76	1.00	1.65
4	0.67	1.94	0.41	0.67


*c*_2_ Adherence to company strategy				
*k*/*l*	1	2	3	4
1	1.00	2.88	0.60	1.48
2	0.35	1.00	0.21	0.52
3	1.65	4.76	1.00	2.45
4	0.67	1.94	0.41	1.00


*c*_3_ Absence of barriers				
*k*/*l*	1	2	3	4
1	1.00	2.88	0.60	1.48
2	0.35	1.00	0.21	0.52
3	1.65	4.76	1.00	2.45
4	0.67	1.94	0.41	1.00


*c*_4_ Maturity of idea				
*k*/*l*	1	2	3	4
1	1.00	2.88	0.60	1.48
2	0.35	1.00	0.21	0.52
3	1.65	4.76	1.00	2.45
4	0.67	1.94	0.41	1.00


*c*_5_ Tech. capacity				
*k*/*l*	1	2	3	4
1	1.00	2.88	0.60	1.48
2	0.35	1.00	0.21	0.52
3	1.65	4.76	1.00	2.45
4	0.67	1.94	0.41	1.00


*c*_6_ Implementation time				
*k*/*l*	1	2	3	4
1	1.00	2.88	0.60	1.48
2	0.35	1.00	0.21	0.52
3	1.65	4.76	1.00	2.45
4	0.67	1.94	0.41	1.00

*Note:* Evaluations of four Projects in *UV* format (see Table 2) converted into six matrices in *MR* format.

Once the evaluation matrix has been constructed, converting the UV format to the MR format, it is possible to apply the AHP method to prioritize the four Projects recovered from the Mapping method and prioritize them within the portfolio formed by recovered and new Projects.

### Prioritization on the basis of AHP

#### Elementary steps of the AHP method: objective, elements, formats, and consistency of evaluations

Saaty's (1980) AHP is operationalized realizing seven steps. This subsection presents the first four steps. First, define the prioritization objective (e.g., suppliers, projects and, stock market). Second, group the elements into criteria, sub-criteria, and alternatives. Third, represent each element through the MR format, that is, compare the elements of each group in pairs using the scale shown in Table 4.

**Table 4:** Fundamental scale of the AHP method [1].

Intensity of Importance	Definition	Explanation
1	Equal importance	Both alternatives contribute equally to the objective.
3	Average importance of one over the other	Experience and judgment lightly favor one alternative in reaction to another.
5	Strong or essential importance	Experience and judgment strongly favor one alternative in reaction to another.
7	Very strong or demonstrated	One alternative is very strongly favored over another; its dominance is demonstrated in practice.
9	Absolutely more important	Evidence favors one alternative over another with the highest degree of certainty.
2,4,6,8	Intermediate intervals	When you are looking for a compromise condition between two definitions.
reciprocal	Use reciprocal for reverse comparison	A reasonable designation.
rational	Reasons resulting from the scale	If the consistency has to be forced to get numeric values n, to complete the array.

Table 5 shows the portfolio of seven Projects with the four evaluated in the UV format and converted to the MR format and three other projects evaluated directly in the MR. Evaluations for criterion $c_{1}$ recovered from UV format are displayed on white cells, and new evaluations in MR format are displayed on green cells.

**Table 5:** MR of $c_{1}$ of the portfolio with Projects evaluated in different formats.

*Note:* The new matrix for the criterion $c_{1}$, based on transforming the corresponding data of Table 3, converting the initial evaluations and including the evaluation of three new projects.

The fourth step is directed at verifying the consistency of the MR matrices. The main advantage of pairwise comparisons is providing the expert with the ability to focus only on two alternatives simultaneously [3]. However, this evaluation limits experts' global perception of alternatives and generates more information than necessary, which leads to inconsistent preferences [18]. Therefore, it is very important to check the consistency of the MR matrices on the basis of the following condition:

(5) $$x_{ij}x_{jk}\;=\;x_{ik}\;\forall i,j,k.$$

When a MR matrix of order n is fully consistent, its maximum eigenvalue ($\lambda_{max}$) is equal to n. When it is inconsistent, $\lambda_{max}\;$exceeds n [19]. If it is used the fundamental scale of Saaty [1], the Consistency Ratio:

(6) $$CR\;=\frac{CI}{RI}$$

can be applied as a measure of inconsistency.

In (6), CI is the Consistency Index calculated as:

(7) $$CI=\frac{\lambda {\;}_{max}-n}{n-1}$$

and RI is the Random Index whose values are given in Table 6.

**Table 6:** Random index.

n	1	2	3	4	5	6	7	8	9	10
RI	0	0	0.52	0.89	1.11	1.25	1.35	1.40	1.45	1.49

*Note:* The Random Index needed to perform the calculation of (6).

If CR≤10%, the matrix is considered acceptable for use [1].

### Ensuring consistency of AHP matrices

Ishizaka and Lusti [20] present an approach to provide the transitivity of MR matrices. This approach consists of rewriting (5) according to the first diagonal higher than the main diagonal of MR. It permits one to present the other elements of the upper part of MR as follows:

(8) $$a_{i,j}\;=\;a_{i,j+1}\;\cdot a_{i+1,i+2}\;\cdot \;\ldots \;\cdot \;a_{j-1,j}.$$

The approach that ensures the consistency of the AHP matrices is performed as follows. First, experts evaluate new Projects directly in the MR format as shown in the green cells of Table 5. Second, the new evaluations' inverse values are recalculated based on the first diagonal higher than the main diagonal of MR. Table 7 shows the results of the recalculation of the inverse values of the MR matrix.

**Table 7:** Recalculation of the inverse values of the MR matrix.

*Note:* Evaluations of Projects in MR format located in the green cells. The main diagonal of the matrix is in the dark gray cells. Inverse values of the evaluations are in the light gray cells. The first diagonal higher than the main diagonal of MR is in the brown cells. Evaluations calculated from the upper diagonal are in the orange cells.

Third, the new evaluations performed at step one are replaced by the inverse values of the evaluations calculated at the second step. For example, $(A_{3},A_{5})\;$is obtained from $\left(A_{3},A_{4}\right)\times \left(A_{4},A_{5}\right)$ and $\left(A_{2},A_{5}\right)$ is obtained from $\left(A_{2},A_{3}\right)\times \left(A_{3},A_{4}\right)\times \left(A_{4},A_{5}\right)$. After applying (8), the Consistency Ratio (CR) of the evaluations for the criteria and alternatives is reduced to zero. That is, the CR of the evaluations before (8): $c_{1}$ (CR=0.05), $c_{2}$ (CR=0.10), $c_{3}$ (CR=0.38), $c_{4}$ (CR=0.03), $c_{5}$ (CR=0.02), and $c_{6}$ (CR=0.05) were reduced to zero, while the maximum threshold for CR acceptance is 0.10.

### Eigenvalue, eigenvector, and ranking of alternatives

This subsection presents the last three steps of applying AHP. Fifth, the calculation of the normalized eigenvector associated with the maximum eigenvalue for each element is realized. There are several ways to calculate $\lambda_{max}$, for instance [21,22].

Sixth, the evaluation of each alternative according to the obtained eigenvector (each element is the respective alternative's weight) is realized as well as the construction of a typical decision matrix [23] (as shown in Table 8) is performed.

**Table 8:** Typical decision matrix [23].

Alts. / Crits.	Weights				
	$\left[\;w_{1},\;w_{2},\ldots ,w_{p},\ldots ,w_{q}\;\right]$				
	*c_1_*	*c_2_*	*c_p_*	*…*	*c_q_*
*A_1_*	*a_11_*	*a_12_*	*a_1p_*	*…*	*a_1q_*
*A_2_*	*a_21_*	*a_22_*	*a_2p_*	*…*	*a_2q_*
*:*	*:*	*:*	*:*	*:*	*:*
*A_n_*	*a_n1_*	*a_n2_*	*a_np_*	*…*	*a_nq_*

*Note:* The decision matrix containing alternatives and weights.

The eigenvector $\left[w_{1},w_{2},\ldots ,w_{p},\ldots ,w_{q}\right]$ is calculated from the comparisons between the considered criteria. This eigenvector is to serve as weights of criteria with the observance of the following conditions: $w_{p}\geq \;0,\;p\;=\;1,2,\ldots ,q\;\text{and}\;\sum_{p=1}^{q}w_{p}=1$. The eigenvectors $[a_{ip}],\;i,p=1,2,\ldots ,q$, p=1,2,…,q are calculated from the comparisons between the alternatives from the standpoint of the criteria $c_{p}$, p=1,2,…,q.

Seventh, the ranking of alternatives is realized, applying the score of each alternative, as follows:

(9) $$y_{i}=\sum_{i=1}^{n}w_{p}a_{ip},\;p=1,\;2,\ldots ,q,$$

where $y_{i}$ is the score of the alternative $i,\;w_{p}$ is the weight of the criterion $c_{p}$, and $a_{ip}$ is the evaluation of the alternative i for the criterion p.

Utilizing (9) for the recovered and new evaluations, the seven Projects in the portfolio were prioritized on the basis of the AHP method. Table 9 presents the portfolio of Projects prioritized applying different preference formats.

**Table 9:** Portfolio of seven Projects prioritized through AHP.

Alts. /Crits.	*c*_1_	*c*_2_	*c*_3_	*c*_4_	*c*_5_	*c*_6_	Score	Ranking
*A*_1_	0.123	0.142	0.029	0.152	0.073	0.056	0.096	5
*A*_2_	0.043	0.049	0.010	0.053	0.025	0.019	0.033	7
*A*_3_	0.204	0.235	0.049	0.251	0.120	0.093	0.158	4
*A*_4_	0.083	0.096	0.020	0.102	0.049	0.038	0.065	6
*A*_5_	0.249	0.096	0.099	0.307	0.147	0.265	0.194	3
*A*_6_	0.249	0.287	0.099	0.102	0.440	0.265	0.240	1
*A*_7_	0.050	0.096	0.694	0.034	0.147	0.265	0.214	2

*Note:* Scores of the seven Projects after applying (9). The ranking is defined by the order of decreasing scores of the Projects.

### Prioritization through the TODIM method

The application of the TODIM method is associated with the following five steps: construction of a decision matrix containing the evaluation of alternatives by criteria; normalization of the evaluations of the decision matrix; building of the normalized weights for the considered criteria; calculation of the degree of dominance between alternatives; and calculation of the degree of general dominance of the alternatives [6,7].

#### Replacing AHP with TODIM

The replacement of AHP by TODIM were carried out in three steps. First, the eigenvectors and normalized weights generated in the AHP [24] were used to build the decision matrix. Second, the normalized value of the “Cost of RD&I Project” was inserted as a seventh criterion in the decision matrix. Therefore, the new matrix (formed by criteria of qualitative and quantitative character) considers the “Cost of RD&I Project” in the prioritization (unlike the Bubble chart that uses the “Cost of RD&I Project” only to define the size of the bubble). Third, projects are prioritized based on calculations of the degree of dominance between the alternatives and the general degree of dominance of the alternatives.

In particular, the degree of dominance of the alternative $A_{i}\;\text{over}\;$the alternative $A_{j}$ is calculated as [6], [7], [8], [9]:

(10) $$\delta \left(A_{i},A_{j}\right)=\sum_{c=1}^{m}\Phi_{c}\left(A_{i},A_{j}\right),\;\forall \left(i,j\right),$$

where

$$\Phi_{c}=\left\{ \begin{array}{c} \sqrt{\frac{w_{cr}\left(P_{ic},P_{jc}\right)}{\sum_{c=1}^{m}w_{cr}}},\;if\left(P_{ic},P_{jc}\right)>0\;\\ 0,\;if\;\left(P_{ic},P_{jc}\right)=0\\ \frac{-1}{\theta }\sqrt{\frac{\left(\sum_{c=1}^{m}w_{cr}\right)\left(P_{jc},P_{ic}\right)}{w_{cr}}},\;if\left(P_{ic},P_{jc}\right)<0 \end{array}\right.$$

In (10), $\Phi_{c}$ represents the contribution of criterion c to the degree of dominance of $A_{i}$ over $A_{j}$;$\;w_{cr}$ is equivalent to $w_{p}$, that is, the weight of the criterion $c_{r}$;$\;P_{ic}$and $P_{jc}$are the performances of the alternatives $A_{i}$ over $A_{j}$ in relation to c;c is any criterion, for c=1,…,m;m is the number of criteria;θ represents the attenuation of loss ratio of criterion c.

It is normally assumed that θ is equal to one, which means that the decision maker has the same degree of aversion to loss in every studied criterion [7].

The general degree of dominance of the alternative $A_{i}$ through normalization of the corresponding dominance degree [6], [7], [8], [9] can be calculated as:

(11) $$\xi_{i}=\frac{\sum_{j=1}^{n}\delta \left(A_{i},A_{j}\right)-\text{min}\sum_{j=1}^{n}\delta \left(A_{i},A_{j}\right)}{\text{max}\sum_{j=1}^{n}\delta \left(A_{i},A_{j}\right)-\text{min}\sum_{j=1}^{n}\delta \left(A_{i},A_{j}\right)}$$

The rank of any alternative is defined by the estimates carried out by applying (11). Thus, after applying (11), it is possible to set the priority of seven projects, taking into account seven criteria, six of which are qualitative and one is quantitative. Table 10 shows the result of this prioritization.

**Table 10:** Portfolio of seven RD&I projects assigned through TODIM method.

Alts. /Crits.	*c*_1_	*c*_2_	*c*_3_	*c*_4_	*c*_5_	*c6*	*c7*	Score	Ranking
*A*_1_	−3,96	−3,71	−3,90	−4,11	−3,95	−4,28	−3,82	−41,0	6
*A*_2_	−4,26	−4,24	−4,21	−4,18	−4,58	−4,72	−4,50	−48,2	7
*A*_3_	−4,19	−4,72	−3,98	−5,06	−3,82	−4,40	−4,38	−20,5	5
*A*_4_	−3,96	−2,81	−3,97	−3,50	−4,14	−4,39	−4,15	−14,3	4
*A*_5_	−4,25	−2,81	−4,17	−5,80	−3,72	−4,18	−5,37	−8,2	3
*A*_6_	−4,25	−5,46	−4,17	−3,50	−7,99	−4,18	−3,81	−5,6	2
*A*_7_	−4,13	−2,81	−10,90	−4,51	−3,72	−4,18	−7,24	−3,3	1

*Note:* Scores of the seven RD&I Projects after applying (11). The ranking is defined by the order of decreasing scores of the Projects.

The comparison of the results of the ranking produced by AHP (Table 9) and by TODIM (Table 10) shows that alternatives *A*_2_ and *A*_5_ maintained their positions. The others five projects moved up or down one position. The inclusion of the Cost of RD&I Projects, which is the criterion of a quantitative character, in the decision matrix explains this change.

## Conclusion

The present paper introduces the “Multimethod to prioritize projects evaluated in different formats”. This method offers flexibility in replacing the project prioritization method, taking advantage of evaluations carried out in diverse preference formats. Such flexibility allows, for instance, companies to keep the prioritization method compatible with their strategy. For example, it can be achieved by adopting the popular and reliable prioritization method such as AHP, or by adopting the method that addresses quantitative criteria and the notion of risk such as TODIM.

## Declaration of Competing Interest

The Authors confirm that there are no conflicts of interest.
